# Feasibility and safety of reprocessing of intracardiac echocardiography catheters for electrophysiology procedures – a large single center experience

**DOI:** 10.1186/s12947-023-00318-4

**Published:** 2023-10-26

**Authors:** Vedran Velagic, Giacomo Mugnai, Ivan Prepolec, Vedran Pasara, Anica Milinković, Andrija Nekić, Jakov Emanuel Bogdanic, Jurica Putric Posavec, Davor Puljević, Carlo de Asmundis, Gian-Battista Chierchia, Davor Milicic

**Affiliations:** 1 University of Zagreb, School of Medicine, Zagreb, Croatia; 2 Department of Cardiovascular Diseases, University Hospital Center Zagreb, Zagreb, Croatia; 3 Electrophysiology and Cardiac Pacing, Division of Cardiology, University Hospital of Verona, Verona, Italy; 4 Heart Rhythm Management Centre, UZ Brussel-VUB, Brussels, Belgium

**Keywords:** Catheter ablation, Intra-cardiac echography, Catheter reprocessing, Environmental impact, Sustainability, Recycling

## Abstract

**Purpose:**

Intra-cardiac echocardiography (ICE) has become an important tool for catheter ablation. Adoption of ICE imaging is still limited because of its prohibitively high cost. Our aim was to study the safety and feasibility of ICE catheters reprocessing and its environmental and financial impact.

**Methods:**

This was a single center retrospective analysis of all consecutive electrophysiology procedures in which ICE catheters were used from 2015 to 2022. In total, 1128 patients were studied (70.6% male, mean age was 57.9 ± 13.2 years). The majority of procedures were related to atrial fibrillation ablation (84.6%)*.*

**Results:**

For the whole cohort, 57 new ICE catheters were used. Consequently one catheter could be used for 19.8 procedures. New catheters were only used when the image obtained by reused probes was not satisfactory. There were no cases of ICE probe steering mechanism malfunction, no procedure related infections and no allergic reactions that could be attributed to the resterilization process. In total, there was 8.6% of complications not related to ICE imaging. Financially, ICE probe reprocessing resulted with 90% cost reduction (> 2 millions of Euros savings for the studied period) and 95% waste reduction (639.5 kg less, mostly non degradable waste was produced).

**Conclusion:**

Our data suggests that ICE catheter reprocessing is feasible and safe. It seems that risk of infection is not increased. Significant economic and environmental savings could be achieved by ICE catheters reprocessing. Furthermore, ICE reprocessing could allow more extensive ICE usage resulting in safer procedures with a potential reduction of serious complications.

**Graphical Abstract:**

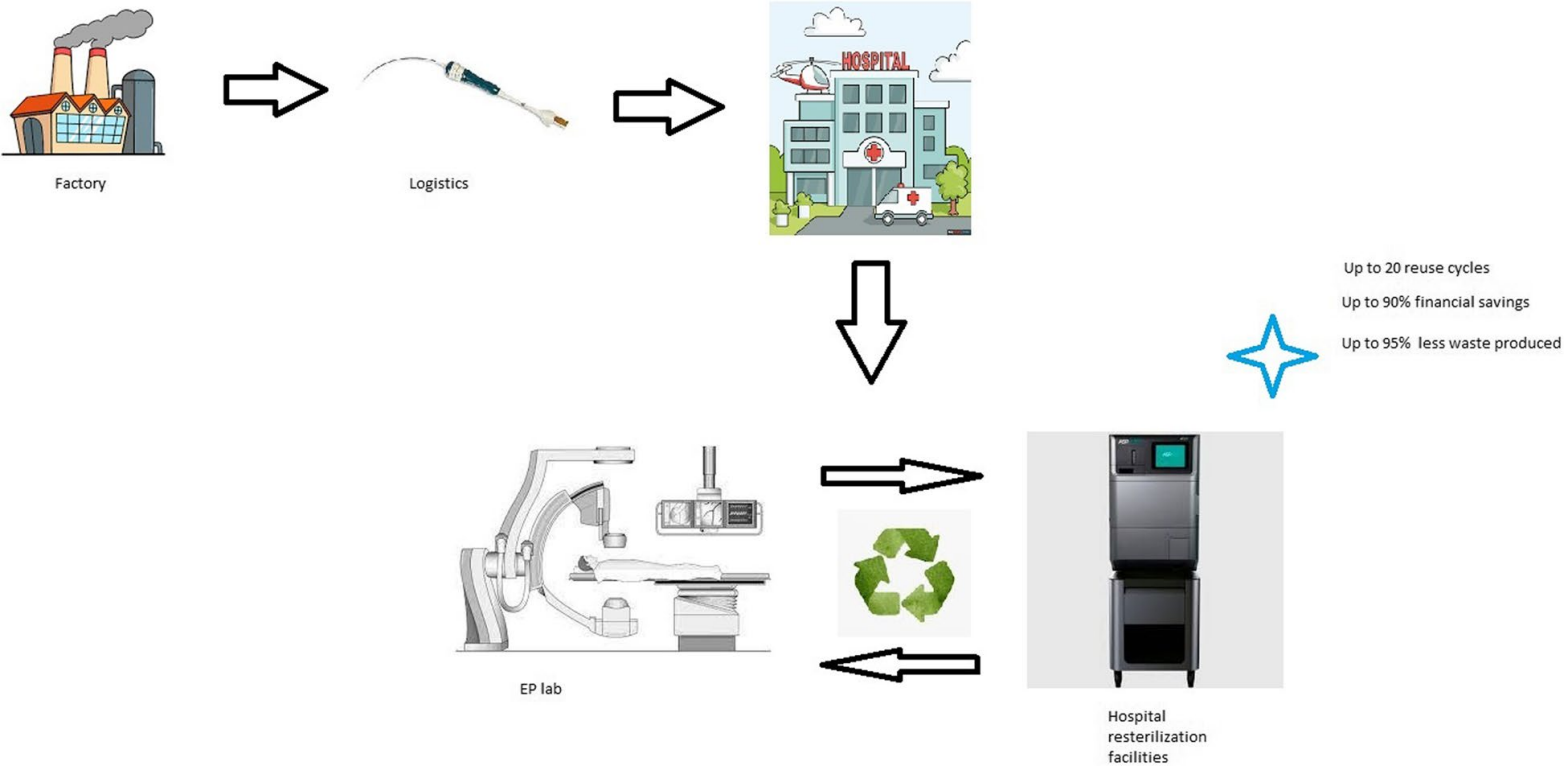

## Introduction

Intra-cardiac echocardiography (ICE) is increasingly used in several percutaneous interventional and electrophysiology (EP) procedures. It has become the essential tool which offers real-time, high-quality evaluation of cardiac anatomy. ICE imaging markedly increases the safety of various cardiac interventions, especially the trans-septal punctures (TSP) and related procedures [1]. Furthermore, ICE is a crucial tool for zero-fluoroscopy interventions [2, 3] and it has the potential to improve the efficacy of EP procedures [4]. Furthermore, ICE catheters are crucial for fluoroscopy reduction in various EP procedures especially for “single shot” devices such as cryoballoon or, more recently, for pulse filed catheters [5].

However, ICE catheters are designated for a single use only and their high cost precludes its widespread adoption. Similar to other EP catheters that are officially single use devices, ICE catheters can be resterilized and reused in several countries that permit reprocessing [6]. ICE resterilization can be beneficial in economic terms, improving the cost effectiveness of EP procedures and allowing safer and more efficient interventions to a higher number of patients. Furthermore, EP material reprocessing could limit unnecessary waste production that is an important part of the “green transition” and greenhouse gas emissions (GHGs) reduction. One potential drawback of ICE reprocessing is a concern about the procedural risks related to material durability and infections. Here we report on a large single center experience about the safety and feasibility of the ICE catheters re-sterilization.

## Methods

### Study population

Data were retrospectively collected from patients having undergone EP procedures using ICE catheters between February 2015 (when we started using ICE imaging) and December 2022 at the University Hospital Centre Zagreb, Croatia. There were no exclusion criteria, and all consecutive procedures when ICE was used were included regardless of arrhythmia and ablation type. Intracardiac echocardiography was consistently used for all atrial fibrillation (AF) procedures to guide TSP. The same was true for all other transseptal procedures including left sided accessory pathway ablations, left sided atrial flutters (Aflu), and structural ventricular tachycardia (VT) ablations. Furthermore, selected typical flutter ablations (such as “redo” cases) and premature ventricular contractions (PVC) ablation cases in specific locations such as aortic cusps and papillary muscles were also performed with the help of ICE. All procedures were performed by 3 different experienced operators and all patients provided written informed consent for the ablation which included the information about catheter resterilization. The research was conducted in accordance with the principles of the Declaration of Helsinki.

### Preprocedural preparation

A transthoracic echocardiogram was performed within six months prior to any ablation procedure to assess the left ventricular ejection fraction and to determine any structural heart and/or valvular disease. A transesophageal echocardiogram was performed before the procedure to exclude left atrial appendage thrombi only in patients who presented with persistent AF or AFlu before procedure. If the patients were anticoagulated, we used the uninterrupted anticoagulation strategy in all patients in accordance with recent recommendations [7].

### Ablation procedures

**Fig. 1:**
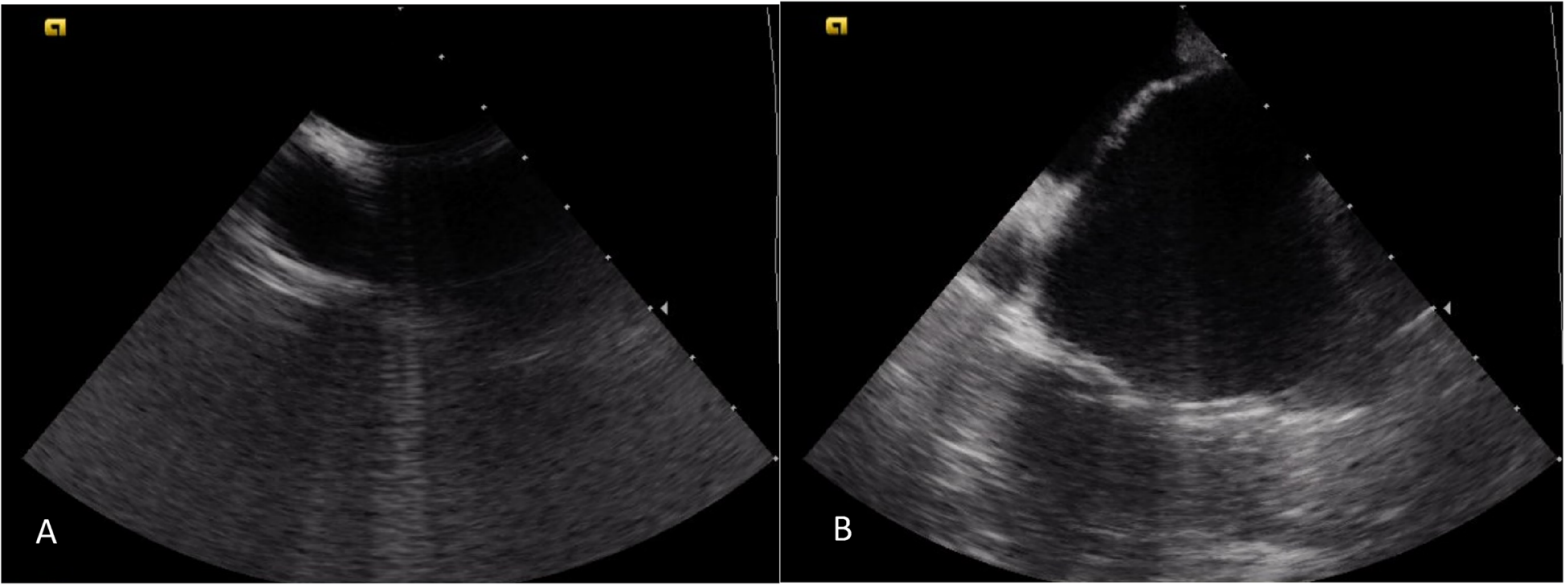
Example of suboptimal intracardiac echo image when the catheter had to be exchanged. **A** ICE image after number of reprocessing cycles **B**) Same image obtained by the new catheter

**Fig. 2:**
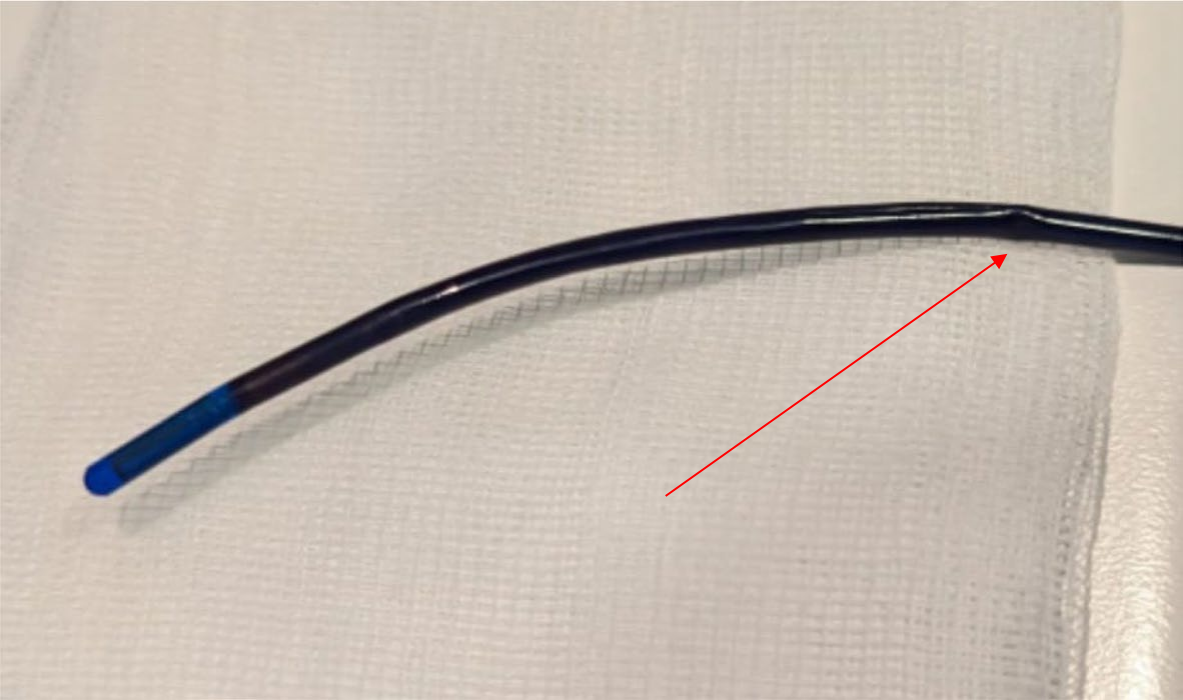
Significant visual damage of the ICE catheter. Please note the kinking of the probe

All procedures were performed under conscious sedation combining fentanyl and diazepam or midazolam. Two types of ICE catheters were used, 8 F and 10 F Accuson Acunav phase array catheters (Siemens AG). For AF ablation, cryoballoon (CB) (Artic Front Advance, Medtronic) was most commonly used. Between 2015–2016 radiofrequency (RF) point by point AF ablations were performed usually with double transeptal punctures using circular mapping catheters. Later on, we adopted a single transeptal puncture strategy with high density (HD) mapping catheters that were exchanged for irrigated, contact sensing ablation catheters. Two different 3D mapping systems were used: Carto 3 (Biosense-Webster) and Ensite Precision (Abbott). Three-D mapping systems were consistently used in all AF: atypical atrial flutter, PVC and VT ablation procedures, except when AF ablation was performed with cryoballoon. In some instances, a 3D mapping system was also used in typical flutter and accessory pathway (AP) ablations, especially after 2019 when we adopted “zero fluoro” strategy for the selected EP procedures. For typical flutters, ICE was most commonly used in redo procedures. For PVC ablations ICE was used in all transeptal procedures and for selected aortic root ablations. Usually, HD mapping catheters were used beside the irrigated contact sensing ablation catheters. For structural VT ablations (ischemic and nonischemic), ICE was consistently used for TSP, guiding the ablation and monitoring for possible complications. Intracardiac echo catheters were always reused while new catheters were opened when acquired images were suboptimal (Fig. 1) or there was significant visual damage to the reprocessed catheter (Fig. 2).

### Postprocedural care and follow up

The possible procedure related infections were assessed by the standard inpatient and outpatient follow-up. Digital hospital records were also scanned. All patients remained in hospital for at least 24 h after the procedure and the follow up visits were scheduled at 3 or 6 months, depending on the type of the procedure. Before discharge, all patients that underwent TSP received bedside transthoracic echo examination to exclude pericardial complications. All patients were advised to come to our emergency room in the case of any possible procedural complications like large access site hematoma, fever, significant chest pain, etc. All possible procedural complications were noted in patient digital hospital records.

### Sterilization procedure

The sterilization was performed in our hospital’s sterilization facilities. We have used the plasma method of low temperature sterilization on the Stearrad NX device (Advanced Sterilization Products). We did not count the exact number or resterilization processes per single catheter. Rather, the catheters were used as long as echo imaging was satisfactory or the catheter did not have clear visible damage to the material. Also, we did not perform any special electrical testing of the catheters. At the beginning of the procedure the resterilized catheter was connected to the echocardiography machine and if the image was present, the catheter was used. In the case that echo machine did not recognize the resterilized catheter, it was discarded.

### Endpoints

The primary endpoint of the study was the safety of the ICE catheter resterilization. We particularly took note of possible infections and allergic reactions related to resterilization. Besides, we searched for all other possible related complications like material embolization or the need for reused catheter extractions (possible “knotting” of reused catheters). Secondary endpoint included the durability of ICE catheters, as we wanted to determine the mean number of resterilization processes before the catheter was deemed unusable.

### Statistical analysis

Categorical variables are expressed as absolute and relative frequencies. Continuous variables followed normal distribution and were expressed as mean ± standard deviation. Statistical analyses were conducted using the SPSS software (SPSS v22, Chicago, IL, USA).

## Results

### Study population

A total of 1128 patients were included in our retrospective analysis. Overall, 70.6% of patients were male and the mean age was 57.9 ± 13.2 years. The majority of patients suffered from atrial fibrillation (84.5%), and atrial flutter was the least common (0.8%). More than half of the population had arterial hypertension (64.4%), and significant portion suffered from heart failure (16.1%). Anticoagulant therapy was highly prevalent in the whole cohort (78.4%). None of the patients had known chronic infections such as hepatitis C virus, hepatitis B virus or human immunodeficiency virus. The detailed baseline clinical characteristics of the study population are provided in Table 1.

**Table 1:** Baseline characteristics

	***N* = 1128**
**Demographic variables**	
Male gender, n (%)	797 (70.6%)
Age at the time of procedure, years (mean ± SD)	57.9 ± 13.2
BMI, kg/m^2^ (mean ± SD)	28.4 ± 4.2
**Main diagnosis**	
Atrial fibrillation, n (%)	954 (84.5%)
Supraventricular tachycardia, n (%)	81 (7.2%)
Atrial flutter, n (%)	9 (0.8%)
Premature ventricular contractions, n (%)	23 (2.1%)
Ventricular tachycardia, n (%)	61 (5.4%)
**Comorbidities**	
Hypertension, n (%)	727 (64.4%)
Diabetes, n (%)	123 (10.9%)
Ischemic heart disease, n (%)	137 (12.1%)
Heart failure, n (%)	182 (16.1%)
Chronic renal failure (eGFR < 60 mL/min), n (%)	87 (7.7%)
Previous TIA/stroke n (%)	57 (5.1%)
**Echocardiography parameters**	
LA diameter, mm (mean ± SD)	40.1 ± 4.2
LVEF, % (mean ± SD)	58.6 ± 10.4
**Anticoagulant/antiplatelet therapy**	
SAPT/DAPT	101 (8.9%)
VKA	143 (12.6%)
DOAC	742 (65.8%)

*BMI* body mass index, *DAPT* dual antiplatelet therapy, *eGFR* estimated glomerular flow rate, *LA* left atrium, *LVEF* left ventricular ejection fraction, *SAPT* single antiplatelet therapy, *TIA* transient ischemic stroke, *VKA* vitamin K antagonists

### Procedural characteristics

For the total of 1128 procedures, 57 new ICE catheters were used. Thirty-four probes were 10 F in size (59.6%) and twenty-three sized 8 F (40.4%). Consequently, one ICE catheter could be used for an average of 19.8 different procedures. There was no case of an ICE probe steering mechanism malfunction, even after multiple reprocessing cycles. We generally used reprocessed catheters, and a new catheter was opened when operator decided acquired images were suboptimal or there was a significant visual damage to the catheter. Vast majority of all interventions included transseptal punctures (99.4%) and all were successfully performed with the help of ICE. Most procedures were AF ablation, and 789/954 (82.7%) of these were performed by the means of cryoballoon ablation. For the remaining procedures, RF energy was used. Three-D mapping system was used in 235 procedures (20.8%). Among them, Carto 3 system was used in 93.1% of cases and Ensite Precision in the remaining 6.9% of procedures. In the whole cohort, mean procedure time and fluoroscopy time were 88.7 ± 47.9 and 11.6 ± 9.4 min, respectively. Acute procedural success was achieved in 97.1% of procedures.

### Complications

There were no procedure-related infections and no allergic reactions which could be attributed to the resterilization process. Furthermore, we did not have cases of material embolization or the need for the special extraction procedures related to resterilized ICE catheters. Also, there were no deaths following the ablation procedures. In total, 97 complications (8.6%) occurred with no correlation with ICE catheters. Vascular complications were the most common, occurring in 42 (3.7%) patients. They included large (> 5 cm) groin hematoma, deep vein thrombosis, arterial pseudoaneurysms and arterio-venous fistulae. The second most common was pericardial effusion without a need of intervention, which was present in 23 (2.0%) patients. There were 8 cases of cardiac tamponade that required drainage (0.7%), 6 cases of pericarditis without sequelae (0.5%) and 13 cases of persistent phrenic nerve palsy (PNP) (1.1%). All PNPs recovered during the follow up and tamponades were treated with pericardial puncture, without a need for surgical intervention. There were 2 cases (0.2%) of gastroparesis that were spontaneously resolved. One case of air embolism was noted (0.1%), one case of mild allergic reaction to protamine (0.1%), and one case of periprocedural stroke (0.1%) related to a VT ablation occurred. The detailed procedural characteristics and complications rates are provided in Table 2.

**Table 2:** Procedural characteristics and complications

	**Total *N* = 1128**	**AF *N* = 954**	**AFlu *N* = 9**	**SVT *N* = 81**	**PVC *N* = 23**	**VT *N* = 61**
Procedure duration (min ± SD)	88.7 ± 47.9	82.3 ± 47.8	77.5 ± 7.7	92.8 ± 45.9	115.2 ± 45.6	198.4 ± 45.7
Fluoroscopy (min ± SD)	11.6 ± 9.4	11.9 ± 9.4	9.0 ± 6.2	8.3 ± 9.3	3.7 ± 9.3	13.6 ± 9.2
**Complications (total)** n (%)	**97 (8.6%)**	**88 (9.2%)**	**1 (1.1%)**	**3 (3.7%)**	**0 (0%)**	**5 (8.1%)**
Vascular, n (%)	**42 (3.7%)**	38 (3.9%)	0 (0%)	2 (2.5%)	0 (0%)	2 (3.3%)
Pericardial effusion, n (%)	**23 (2.0%)**	22 (2.3%)	1 (11.1%)	0 (0%)	0 (0%)	0 (0%)
PNP, n (%)	**13 (1.1%)**	13 (1.4%)	0 (0%)	0 (0%)	0 (0%)	0 (0%)
Tamponade, n (%)	**8 (0.7%)**	6 (0.6%)	0 (0%)	0 (0%)	0 (0%)	2 (3.3%)
Pericarditis, n (%)	**6 (0.5%)**	6 (0.6%)	0 (0%)	0 (0%)	0 (0%)	0 (0%)
Air embolism, n (%)	**1 (0.1%)**	0 (0%)	0 (0%)	1 (1.2%)	0 (0%)	0 (0%)
Protamine allergy, n (%)	**1 (0.1%)**	1 (0.1%)	0 (0%)	0 (0%)	0 (0%)	0 (0%)
Stroke, n (%)	**1 (0.1%)**	0 (0%)	0 (0%)	0 (0%)	0 (0%)	1 (1.6%)
Gastroparesis, n (%)	**2 (0.2%)**	2 (0.2%)	0 (0%)	0 (0%)	0 (0%)	0 (0%)

### Economic and environmental outcomes

New ICE catheter cost in our institution was 2208.84 Euros. If we had always used new catheters for 1128 procedures that would have accounted for 2,491,571.52 Euros in total. On the other hand, catheter resterilization cost was 103.50 Euros per catheter. We resterilized each ICE probe for almost 19 times which adds up to 110,848.50 Euros. Therefore, cumulative cost (new catheters + resterilization cost) of ICE imaging in our institution in the aforementioned period was 236,752.38 Euros. Hence, ICE probe reprocessing resulted with the total savings of 2,254,819.14 Euros in nearly 8 years. Furthermore, one whole ICE catheter package weights 596 g. In total, by reprocessing, 639.5 kg less waste was produced. The ICE catheter itself weights 130 g and it mainly consist of synthetic, nondegradable materials. Therefore, in that time frame, 139.2 kg less, complex, non-degradable medical waste was produced.

## Discussion

### The main findings

The main findings of this study are i) one ICE catheter could be used for up to 20 different EP procedures, ii) ICE catheter reprocessing does not result in increased risk of infections or possible other resterilization-related complications, iii) significant economic and environmental savings could be achieved by ICE catheter reprocessing.

### The impact of EP catheter reprocessing

There are two different advantages of EP/ICE catheters reprocessing that have to be taken into account. Firstly, catheter reprocessing could allow higher number of safer and more efficient ablation procedures for the growing number of patients. Secondly, material reprocessing might significantly limit the negative environmental impact of healthcare industry [8]. Worldwide, the health sector is responsible for up to 4.6% of total GHGs emissions. Data from the United States, where the share is 8.5%, show that health care system is becoming more, not less, polluting.

Cardiac electrophysiology is a medical field with a heavy hi-tech burden, characterized by single use tools that consist of plastics, common and rare metals, microchips and circuit boards that are discarded after only few hours of use (or even less). This practice is high environmentally unfriendly and unsustainable, especially when one takes in to the account that more than 1,100,000 EP procedures are performed yearly worldwide and the numbers are expected to grow [9]. Thus, cardiac EP generates large amounts of highly complicated waste and reducing it’s the environmental impact has become the serious challenge [10, 11]. In our study, we have showed that by ICE catheter reprocessing we could lower the amount of complex medical waste related to intracardiac echo imaging by 95% (form 146.6 kg to 7.4 kg) in the studied period. Most probably, comparable or even larger ecologic impact could also be achieved for other types of EP catheters reprocessing.

Similar to other surgical tools, until 1980 EP catheters were customarily re-used in the United States. Later on, the new regulation was passed that required that manufacturers provide evidence of reusability or classify the device as disposable [12]. New legislation resulted that almost 100% of EP catheters nowadays are declared single use only, despite the fact that in the real world, they can and are being reused. Current manufacturing process of the ICE probes results with a high catheter sturdiness and durability. Four-directional steering mechanism practically never fails, even after a number of resterilization cycles. Because ICE probes typically do not have a lumen, our institution legally allows such process. Surely, the quality of acquired echo images declines with each reuse (Fig. 1), but generally, obtained images allow adequate visualization of the trans-septal system and a safe trans-septal puncture. The EP catheter resterilization process seems to be safe and efficient as demonstrated by a reprocessing validation study by Lester et al. By using total organic carbon determinations, they showed that detergent residues on reprocessed used catheters were nominal and significantly lower than organic carbon levels present in new catheters [13]. Accordingly, in our study we did not find any cases of infections or allergic reactions related to the resterilization procedure. Sterility is surely a major concern. However, a similar large study with resterilized pacemakers and defibrillators did not find excess risk of infection or total mortality in patients with resterilized devices over a 2-year period of follow up [14]. This suggests that from the infection point of view, cardiac device resterilization seems quite safe.

Reprocessed EP catheter functionality is another issue. There is a number of older studies that showed reprocessed EP catheters are safe and functional if routine visual inspection, electrical continuity testing, and confirmation of manual deflection ability are employed [15–17]. A more recent study showed that diagnostic EP catheters can go through five use/reprocessing cycles without measurable loss of functionality [18]. Moreover, our study suggest that ICE catheters are even more durable than diagnostic EP catheters. They might undergo up to 20 resterilization processes before they lose its functionality. Even repeated resterilization process does not result with the major loss of imaging quality and steering ability. Furthermore, probably because of the high material durability, we did not have cases of material embolization or the need for catheter extraction procedures even after high number of resterilization procedures.

Reprocessing of ICE probes could lead to a higher percentage of transeptal punctures performed with the help of ICE imaging, consequently increasing the safety of the procedures, especially when considering cardiac tamponade. In a large German AF ablation registry that included more than 20000 patients, the overall rate of tamponade that required intervention was 0.9%. The proportion of patients with tamponade went up to 2% in low volume centers that used RF energy. The exact proportion of ICE guided TSP was not reported in that study [19]. Catheter reprocessing is allowed in Germany but generally, ICE usage is not very common [20]. In our cohort of AF patients, the tamponade rate was only 0.6%. Furthermore, we did not record a single case of aortic root puncture which could potentially lead to cardiac surgery. The low rates of these potentially devastating complications could be attributed to the consistent use of ICE imaging to guide TSP. It is important to emphasize that because of financial constraints, we would not be able to use a new ICE probe for every procedure and one might hypothesize that it would lead to higher number of transseptal puncture related complications. One possible drawback of ICE catheter use is the potential of a higher number of vascular complications because of the need for an extra vascular access. However, for vast majority of our cases, an extra venous puncture was not required because ICE catheter was replaced for other EP catheter after the transeptal puncture (e.g., ICE catheter was exchanged for a decapolar EP catheter for phrenic nerve pacing after transeptal puncture in all cryoballoon procedures).

Financially, ICE probe reprocessing resulted with more than 90% of cost reduction for intracardiac imaging. Similar economic effects were already reported for EP and other cardiac single use devices [21, 22]. By this means we were able to offer safer procedures to a higher number of our patients. Most commonly, reimbursement is the main obstacle for widespread use of ICE catheters, especially in Europe. In many ways, legislation in European Union (EU) is not harmonized. Some EU countries like Portugal, Norway, Netherlands, Belgium, Germany, Croatia and Bulgaria allow reprocessing. However, in Germany reprocessing of ICE catheters is limited by the expiration date of the original product. Conversely, in Spain, France, Denmark, Italy, Chechia and Poland currently it is illegal to reprocess the catheters [20]. In the times of heightened financial constraints, the concept of ICE reprocessing would lead to a more extensive use of the ICE in EU resulting in safer procedures with a potential reduction of serious complications. Recently, there are various initiatives for the increase of EP material reprocessing [6]. In the recent European physician survey majority of the respondents are motivated to reduce environmental impact of cardiac electrophysiology and do support EP material reprocessing [20]. Nowadays, some of the companies started producing multiple use catheters [23] and novel circular business models are being developed to reduce the detrimental impact of health care to the environment [24].

### Limitations

This was a retrospective, single center study with all inherent limitations of this type of research. The number of studied procedures with resterilized ICE catheters was significant but we might miss some very rare complications that might occur in < 1:1000–10000 procedures. Although we advised all patients to come to our ER in the case of any possible complication of ablation procedures, some complications might be missed. Because of the retrospective nature of the study, we could have also missed some procedure-related infections. Furthermore, we do not have the exact count of resterilization processes per one particular catheter, rather, we have calculated the mean resterilization number for all catheters in total. Still there is a significant number of countries in EU that does not allow resterilization. Furthermore, even the counties that allow resterilization have different reprocessing policies. This significantly limits the generalizability of our study.

## Conclusion

Our data suggests that ICE catheter resterilization and reuse is feasible and safe. Good quality of ICE probe manufacturing allows a high number of reuse cycles. It seems that it does not increase the risk of infection or allergic reactions. Significant economic savings could be achieved by ICE catheters reprocessing and even more important is the potential to reduce negative environmental impact of cardiac electrophysiology. Moreover, ICE reprocessing could increase the proportions of ICE-guided EP procedures worldwide which might result in a lower number of serious procedure-related complications.

## Data Availability

No associated data.
